# International Care Bundle Evaluation in Cerebral Hemorrhage Research (I-CATCHER): Study protocol for a multicenter, batched, parallel, cluster-randomized trial with a baseline period

**DOI:** 10.1177/17474930251342888

**Published:** 2025-05-12

**Authors:** Trine Apostolaki-Hansson, Menglu Ouyang, Dar Dowlatshahi, Valeria Caso, Alessandro Bufi, Zhe Kang Law, Laurent Billot, Bo Norrving, Craig S Anderson, Teresa Ullberg

**Affiliations:** 1 Department of Neurology, Skåne University Hospital, Malmö, Sweden; 2 Department of Clinical Sciences Lund, Lund University, Lund, Sweden; 3 The George Institute for Global Health, Faculty of Medicine, University of New South Wales, Sydney, NSW, Australia; 4 Department of Medicine (Neurology), Ottawa Hospital Research Institute, University of Ottawa, Ottawa, ON, Canada; 5 Stroke Unit, Department of Emergency and Vascular Medicine Santa Maria della MIsericordia Hospital, University of Perugia, Perugia, Italy; 6 Department of Medicine, Faculty of Medicine, National University of Malaysia (UKM), Kuala Lumpur, Malaysia; 7 Institute of Science and Technology for Brain-Inspired Intelligence, Fudan University, Shanghai, China

**Keywords:** Intracerebral hemorrhage, care bundle, blood pressure lowering, cluster trial, implementation, outcome

## Abstract

**Rationale:**

A care bundle approach to the management of spontaneous intracerebral hemorrhage (ICH) has been shown to benefit patients in low- and middle-income countries (LMIC), but uncertainty persists over the specific components and its applicability in high-income countries (HICs).

**Aims:**

An international collaborative initiative aimed at determining whether implementation of a care bundle improves functional outcome for patients with ICH in HIC.

**Methods:**

An international, multicenter, batched, parallel, cluster-randomized clinical trial focused on implementation and quality improvement for adults with spontaneous ICH ⩽ 24 h of symptom onset. The care bundle includes time- and target-based interventions: early intensive blood pressure lowering, hyperglycemia and pyrexia management, anticoagulation reversal, avoidance of do-not-resuscitate orders, repeat imaging, and referral pathways for intensive care and neurosurgery. An embedded process evaluation will assess the effectiveness and implementation of the care bundle.

**Sample size:**

A total of 110 hospitals with 3500 ICH participants is estimated to provide 90% power (α = 0.05) to detect a plausible treatment effect of 0.20 improvement in utility-weighted modified Rankin scale (UW-mRS) scores.

**Outcomes:**

The primary outcome is UW-mRS at 6 months. Secondary outcomes include death, functional status, and health-related quality of life. Implementation outcomes include adoption, fidelity, acceptability, sustainability, and integration.

**Discussion:**

We aim to provide reliable evidence to accelerate practice change for integration of a multifaceted ICH care bundle as a critical component of acute stroke care worldwide.

**Trial registration:**

Clinicaltrials.gov Identifier: NCT06429332.

## Graphical abstract

**Graphical abstract:**
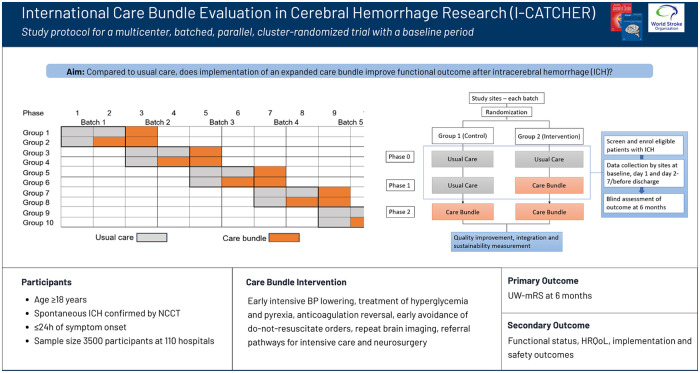

## Introduction

Spontaneous intracerebral hemorrhage (ICH) accounts for up to 15% of acute strokes in high-income countries (HICs), but over half of all new stroke-related deaths and disability worldwide.^1^ Although a decreasing incidence of ICH has been observed in some countries, the burden of ICH is set to increase in Europe and other countries over the coming decades due to the aging of populations and lifestyle changes.^2^

The lack of proven treatments for ICH has created a sense of pessimism in many physicians, variable uptake of guideline-recommended treatments, and unnecessary use of early withdrawal of care in these patients.^3^ The devastating effects of ICH and the scarcity of randomized evidence to support single interventions have produced a nihilistic approach and over-estimates of adverse outcomes.^4^ Several prognostic variables in ICH include hematoma volume,^3^ level of consciousness,^5^ intraventricular hemorrhage,^6^ degree of hematoma expansion,^7^ and use of antithrombotic drugs.^8^ Management strategies that aim to limit hematoma growth include early intensive blood pressure (BP) lowering, reversal of anticoagulation, management of hyperglycemia, and targeted neurosurgery that includes the use of minimally invasive surgery (MIS),^9^ decompressive hemicraniectomy,^10^ and other approaches.

While efforts continue to define the role of individual interventions, evidence has emerged of the benefits of bundles of care in ICH.^3,11^ In particular, the third INTEnsive care bundle with blood pressure Reduction in Acute Cerebral hemorrhage Trial (INTERACT3) showed that the implementation of a systems of care approach that comprised a care bundle protocol for intensive BP lowering and other management algorithms for physiological control within several hours of ICH resulted in improved functional outcome.^11^ However, as most of the 121 hospitals which participated in INTERACT3 were located in low- and middle-income countries (LMICs), and the care bundle was dominated by intensive BP lowering, there may be uncertainty over the effectiveness, specific components, and fidelity of implementation of a care bundle in HIC.

Herein, we outline the protocol for the International Care Bundle Evaluation in Cerebral Hemorrhage Research (I-CATCHER) study that aims to evaluate the effectiveness of a relevant multifaceted care bundle for patients with ICH compared to usual standard care in hospitals located in HICs and some LMICs.

## Methods

### Study design

**Figure 1.**
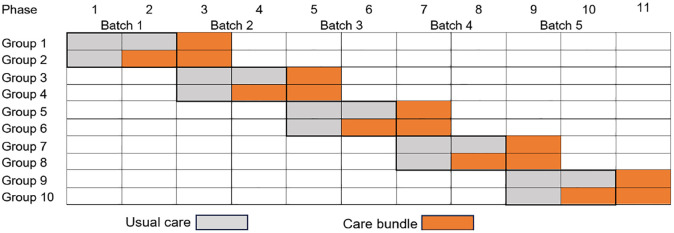
Parallel cluster-randomized design with a baseline period. Participating sites are launched in batches according to readiness. A group represents the number of sites in each region that are cluster-randomized to either the intervention or the control arm.

**Figure 2.**
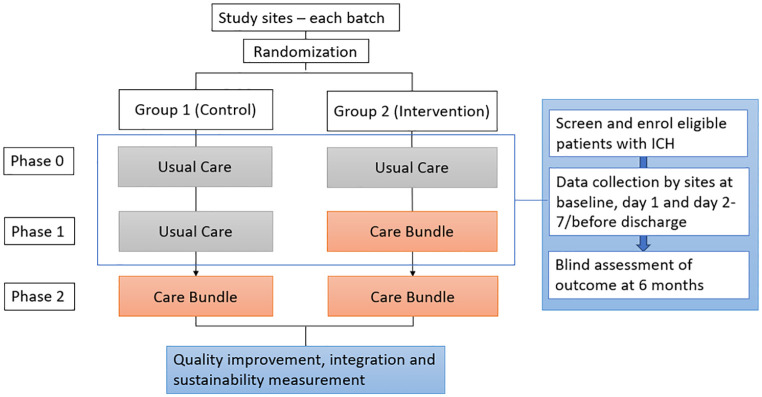
Study schema.

I-CATCHER is an international, multicenter, batched, parallel, cluster-randomized clinical trial with a baseline usual care period to evaluate a care bundle against current standard care in patients with spontaneous ICH. Participating sites are activated in batches, including a pre-determined size of 10 sites, allowing for consecutive enrollment of participants as additional hospitals join (Figure 1). Hospitals within a batch are randomized into two groups—intervention or control arm. The trial includes three phases: a baseline usual care phase; a randomized intervention phase; and a post-implementation period, once all sites have switched to the intervention (Figure 2). All participants are followed up for 6 months. Only data from the baseline and randomized phases will be used for the primary analysis. This study uses a type I hybrid design, which allows the simultaneous evaluation of the effectiveness of the intervention as well as its implementation in a broad range of healthcare settings.

### Population

The trial will include adults (age ⩾18 years) who present with spontaneous ICH. The inclusion and exclusion criteria are outlined in Table 1.

**Table 1.** Inclusion and exclusion criteria.

Inclusion criteria
• Adults (age ⩾ 18 years)
• NCCT or MRI-verified diagnosis of spontaneous ICH
• ⩽24 h from symptom onset or last seen well
Exclusion criteria
• Previous care limitation (i.e. previous DNR orders and/or limitations on life-sustaining treatment in an ICU setting, including any written patient directives regarding these limitations)
• End-stage comorbidity with short life-expectancy (<6 months, e.g. terminal cancer)
• ICH caused by brain tumor or cerebral venous thrombosis
• Clinical signs of brain herniation at first presentation
• Pregnant women beyond 22 gestational weeks may only be included after a thorough risk–benefit discussion with an obstetrician

DNR: do-not-resuscitate, ICH: intracerebral hemorrhage, ICU: intensive care unit, NCCT: non-contrast computerized tomography, MRI: magnetic resonance imaging.

### Randomization and blinding

An independent statistician will assign hospitals in a 1:1 ratio to the care bundle or usual care. Within each batch, participating sites will be stratified according to country and level of site (comprehensive tertiary with neurosurgeon services vs. secondary hospital without neurosurgeon services). Sites will be notified of their randomized group within 2 weeks of an agreed date before commencing the intervention. Treatment allocation is open label.

### Intervention

Implementation strategies will be used to introduce an active care bundle with time- and target-based metrics. Table 2 lists the components of the care bundle.

**Table 2.** Components of the care bundle intervention.

1. Reversal treatment of ongoing OAC, with either: 1. An elevated INR with the use of warfarin—treatment ⩽ 30 min after ICH detection and maintain INR target < 1.3; or 2. Use of a DOAC—treatment ⩽30 min with direct/indirect reversal agent
2. Early intensive BP lowering. 1. Systolic BP < 200 mm Hg after ICH detection: systolic target of 130–140 mm Hg should be achieved ⩽ 30 min of commencing treatment and maintained for the first 7 days. 2. Systolic BP ⩾ 200 and < 220 after ICH detection: systolic target of 160 mm Hg should be achieved ⩽ 30 min, and 130–140 mm Hg should be achieved ⩽ 60 min and maintained for the first 7 days. 3. Systolic BP ⩾ 220 after ICH detection: systolic target of 160 mm Hg should be achieved ⩽ 60 min and maintained for the first 7 days.
3. Pyrexia treatment: achieve a body temperature target < 37.5°C for 24 h from ICH detection
4. Hyperglycemia treatment: maintain a blood glucose level between 7 and 10 mmol/L for 24 h from ICH detection
5. No use of DNR or withdrawal of care for 48 h
6. Immediate (< 30 min) *referral* for intensive care if airway, breathing, and/or circulation are compromised during hospital stay (events related to the first 7 days of hospital stay are recorded in the eCRF)
7. Immediate (< 30 min) *referral* to neurosurgery if any of the following: 1. Large and/or rapidly evolving supratentorial ICH (> 20 ml volume) 2. Any IVH extension 3. Posterior fossa bleed, irrespective of volume 4. Suspicion of a vascular malformation, independent of volume or location 5. Altered consciousness or neurological deterioration since symptom onset or during hospital stay if related to ICH (events related to the first 7 days of hospital stay are recorded in the eCRF)
8. Repeat 6–12 h brain imaging if there is clinical deterioration or if the patient received OAC reversal treatment

BP: blood pressure, °C: degrees centigrade, DOAC: direct oral anticoagulant, DNR: do-not-resuscitate, ICH: intracerebral hemorrhage, INR: international normalized ratio, IVH: intraventricular hemorrhage, mmol/L: millimole per liter, OAC: oral anticoagulant.

For participants in the usual care group, decisions regarding their investigations, monitoring, and management are made by the attending clinical team.

### Clinical outcomes

The primary outcome is the utility-weighted modified Rankin scale (UW-mRS) score assessed at 6 months (180 ± 30 days). Secondary clinical efficacy outcomes will also be assessed at 6 months: (1) ordinal shift analysis of mRS; (2) death (mRS 6); (3) disability (mRS 3–5); (4) poor outcome (mRS 3–6); (5) health-related quality of life (HRQoL) based on the EuroQol 5-Dimension (EQ-5D); and (6) any readmission to hospital. Safety outcomes are (7) all serious adverse events (SAE) up to the study end; (8) SAEs ⩽ 7 days: episodes of severe hypotension (< 100 mm Hg) or hypoglycemia (< 4 mmol/L); and (9) SAE ⩽ 30 days: occurrence of any venous or arterial thromboembolic event. An SAE is defined as death; significant deterioration in a participant’s health characterized by a life-threatening illness or injury related to the study intervention (as stated above).

Centralized follow-up of participants will be undertaken by independent research staff blinded to treatment allocation. Follow-up clinical outcome assessors will contact the participant or a relative by telephone at 6 months. A health economic evaluation will be performed after study closure.

### Implementation outcomes

**Figure 3.**
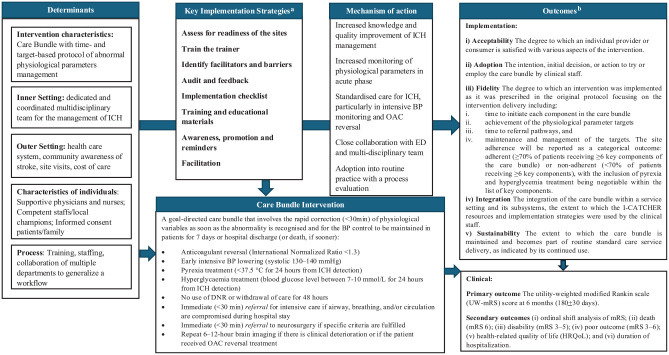
Logic model illustrating the implementation process and its relationship to clinical and implementation outcomes. ^a^Supplemental Table 2 for more details on key implementation strategies. ^b^Supplemental Table 1 for more details on implementation outcomes.

The implementation outcomes are acceptability, fidelity, adoption, integration, and sustainability (Supplemental Table 1). To assess implementation outcomes, a prospective process evaluation (PE) will be embedded in the trial that uses a mixed-methods approach derived from audit data, implementation checklists, surveys, and semi-structured interviews (Supplemental Table 2). The PE will be guided by the Medical Research Council framework for evaluating complex interventions at an early phase to identify barriers and facilitators to improve implementation of the care bundle.^12^ Figure 3 illustrates a logic model describing the implementation process.

## Data safety monitoring board

An independent data safety monitoring board (DSMB) will review the safety and outcomes of the study, the quality of the trial conduct, monitor protocol adherence, and provide feedback to the Steering Committee on the conduct of the study as appropriate. The DSMB will meet 6-monthly to monitor the study progress and safety. A formal interim analysis will be performed by an independent statistician, blind to the randomized allocation, after 50 sites have reached data maturity. These data will then be reported to the DSMB for review of participant safety and outcomes as well as the quality of trial conduct. The DSMB will monitor unblinded safety outcomes and provide reports to the Steering Committee and recommendations in relation to continuation or stopping the study. A detailed DSMB charter will be made available that specifies the safety monitoring procedure and stopping rules.

## Statistical considerations and methods

### Sample size calculation

This study is designed with 90% power (α = 0.05) to detect an effect size of 0.20 improvement in the mean score of the UW-mRS, which corresponds to a mean difference of 0.07 (standard deviation = 0.356) between the care bundle group and the standard care group. The sample size is based on the INTERACT3 trial, in which the mean difference in UW-mRS scores between usual care (0.58) and care bundle (0.62) groups was 0.04.^11^ Considering the inclusion of components of anticoagulant reversal as well as referral pathways to intensive care and neurosurgery, we assume the effect of the care bundle in I-CATCHER will be greater than in INTERACT3.

Recruitment of a minimum of 110 sites is anticipated. To demonstrate a treatment effect with 90% power and a two-sided type I error rate of 5%, each site will need to recruit an average of 15 participants per phase for a total sample size of 3300 patients (110 sites × 2 phases × 15 participants). Assuming 5% of participants will have a missing 180-day outcome data, a total of 3500 participants are required which corresponds to a mean of 16 participants recruited per period per hospital. These calculations assume a within-period intra-cluster correlation (ICC) of 0.05 and a two-period decay with a cluster autocorrelation of 0.8 which are consistent with data from the INTERACT3 and Head Position in Acute Stroke Trial. Calculations were performed using the Shiny CRT Calculator.^13^

### Statistical analysis

All analyses will be undertaken at the patient level on an intention-to-treat basis using a linear mixed model with a random site effect to account for within-cluster correlations. The model will include fixed effects for the batch as well as stratification variables (i.e. country and level of site) and will adjust for data collected during the baseline period as suggested by Hooper et al.^14^ The effect of the intervention will be estimated as the adjusted mean difference and 95% confidence interval. Secondary outcomes will be analyzed using a similar approach but with logistic regression for binary outcomes and assuming a proportional odds model for the ordinal analysis of mRS. A detailed analysis plan including mock tables will be finalized before unblinding and made publicly available.

## Consent procedures

The consent process follows two processes:

1. Hospital cluster consent: Sites will consent to participate in endorsing the Care Bundle as standard practice for managing patients with ICH.
2. Patient/responsible informed consent: Consent is obtained for the registration of patient data in the eCRF and participation in follow-up. A next of kin will be consulted for patients unable to provide consent. The consent process may vary by country, depending on the decisions made by their Institutional Research Ethics Committee.

## Discussion

I-CATCHER aims to evaluate the effectiveness of a multi-component care bundle for spontaneous ICH compared to standard care across the full spectrum of ICH severity. To better reflect the broad range of characteristics of patients affected by ICH, I-CATCHER features broad inclusion criteria, narrow exclusion criteria, and an efficient consent process. These factors facilitate the rapid inclusion of the vast majority of ICH patients into the study, enhancing both the generalizability of the study and the rate of recruitment. The trial design will capture consecutive patients with ICH and allow continued intervention as more hospitals join. Unlike a conventional stepped-wedge cluster randomized controlled trial (RCT), the intervention effect in this design reduces the risk of confounding by background temporal trends, as only baseline and parallel comparison data are used to assess the effectiveness of the care bundle.

Alongside its randomized design, I-CATCHER has a clear focus on implementation science through the determination of clinical efficacy of the care bundle but also by enabling its sustainable integration into routine practice across all participating hospitals. Previous studies, including a clinical trial involving a similar care bundle intervention in LMIC, have demonstrated significant patient benefit. However, patient demographics varied considerably from those in HIC. I-CATCHER extends this investigation to HIC with established ICH protocols, aiming to determine whether a time- and target-based intervention, combined with direct referral to neurosurgery and intensive care, can further improve patient outcomes in these settings.

## Summary and conclusion

As the first international RCT comparing an ICH care bundle intervention with standard care in HIC, I-CATCHER seeks to provide critical insights into whether this approach can improve 6-month outcomes for ICH patients. If successful, I-CATCHER could lead to changes in future ICH guidelines and potentially lead to widespread adoption of the ICH care bundle intervention, offering significant benefits to a patient population that currently experience high rates of mortality and morbidity.

## Supplemental Material

sj-docx-1-wso-10.1177_17474930251342888 – Supplemental material for International Care Bundle Evaluation in Cerebral Hemorrhage Research (I-CATCHER): Study protocol for a multicenter, batched, parallel, cluster-randomized trial with a baseline period Supplemental material, sj-docx-1-wso-10.1177_17474930251342888 for International Care Bundle Evaluation in Cerebral Hemorrhage Research (I-CATCHER): Study protocol for a multicenter, batched, parallel, cluster-randomized trial with a baseline period by Trine Apostolaki-Hansson, Menglu Ouyang, Dar Dowlatshahi, Valeria Caso, Alessandro Bufi, Zhe Kang Law, Laurent Billot, Bo Norrving, Craig S Anderson and Teresa Ullberg in International Journal of Stroke
