# Supplementary material for: International Care Bundle Evaluation in Cerebral Hemorrhage Research (I-CATCHER): Study protocol for a multicenter, batched, parallel, cluster-randomized trial with a baseline period
Source: Int J Stroke. 2025 May 12;20(7):891–7. doi: 10.1177/17474930251342888 (PMC12264302; doi:10.1177/17474930251342888)
Supplement: sj-docx-1-wso-10.1177_17474930251342888 – Supplemental material for International Care Bundle Evaluation in Cerebral Hemorrhage Research (I-CATCHER): Study protocol for a multicenter, batched, parallel, cluster-randomized trial with a baseline period [file sj-docx-1-wso-10.1177_17474930251342888.docx]

**Supplemental Material: Tables**

| **Supplemental Table 1. Description of implementation outcomes** | |
| --- | --- |
| Acceptability | The degree to which an individual provider or consumer is satisfied with various aspects of the intervention (such as content, complexity, comfort, delivery, and credibility). Measured early for adoption from survey and semi-structured interviews. |
| Fidelity | The degree to which an intervention was implemented as it was prescribed in the original protocol. In this study, the measurement of fidelity is focusing on the intervention delivery including:   1. time to initiate each component in the care bundle 2. achievement of the physiological parameter targets 3. time to referral pathways, and 4. maintenance and management of the targets. The site adherence will be reported as a categorical outcome: adherent (defined as ≥70% of patients receiving at least 6 key components of the care bundle) or non-adherent (defined as <70% of patients receiving at least 6 key components). This adherence classification will be based on accepted implementation targets, with the inclusion of pyrexia and hyperglycemia treatment being negotiable within the list of key components. |
| Adoption | The intention, initial decision, or action to try or employ an innovation or evidence-based practice. The intention and uptake of the care bundle by clinical staff. Measured from staff survey and semi-structured interviews. |
| Integration | The integration of the care bundle within a service setting and its subsystems, the extent to which the I-CATCHER resources and implementation strategies were used by the clinical staff. Measurements are from audit data, implementation checklist, and non-participant observation. |
| Sustainability | The extent to which the care bundle is maintained and becomes part of routine standard care service delivery, as indicated by its continued use. Measurements are derived from audit data, implementation checklists, and non-participant observation. All sites will be asked if they are going to continuously deliver the care bundle after the implementation phase (phase 2). If they agree, a follow up audit will be conducted at three-month intervals for one year to assess the sustainability of the intervention’s implementation. An additional sub-study will focus on integrating the intervention metrics into the Swedish Stroke Register to support long-term quality improvement in stroke care. |

| **Supplemental Table 2. Implementation strategies and data collection methods** | |
| --- | --- |
| **Strategy** | **Descriptor** |
| Assess for readiness of the sites | Data collection method using a survey-based feasibility questionnaire (implementation checklist) to provide to sites in phase 0 to assess various aspects of an organisation to determine its degree of readiness to implement, barriers that may impede implementation, and strengths that can be used in the implementation effort of the ICH care bundle. |
| Train the trainer | Using a train the trainer approach at site visits: designated clinicians will train others in applying the ICH care bundle intervention. |
| Identify facilitators and barriers | Semi-structured interviews with physicians and nurses. *Process evaluation interviews* including open-ended questions using the normalization process theory to optimize the implementation process. |
| Audit and feedback | Data on clinical performance related to the ICH care bundle interventions and adherence to protocol during the baseline period will be collected and analyzed throughout the trial. Monthly feedback will be provided to sites to inform, monitor, evaluate, and improve implementation of the ICH care bundle. During the intervention phase, sites will attend at least two on-line or in-person quality improvement meetings in performance is suboptimal. |
| Implementation checklist | A bedside document will act as source data for key features in the Case Report Form that are believed to be lacking in the patient medical records. |
| Training and educational materials | Training will be provided to sites including training videos for the separate interventions with evidence-based recommendations, pocketcards with eligibility criteria and recommendations on how to achieve intervention targets, a standardized reporting protocol for radiologists will be provided based on radiological imaging variables present in the Case Report Form; Sweden: on-call 24 hour service for questions regarding eligibility. These materials will be made available to participating sites to enhance implementation of the ICH care bundle with the intent of changing practice through skill acquisition. |
| Awareness, promotion and reminders | Reminders and alerts will regularly be sent through email designed to help clinicians and study personnel stay motivated and prompt them to continue recruiting patients into the study, collecting data, and employing the ICH care bundle. |
| Facilitation | A process of interactive problem solving and support that occurs in a context of a recognised need for improvement and a supportive interpersonal relationship. This will be offered throughout the course of the study by online meetings with the national coordinating teams and principal investigators. |
| Abbreviations: *ICH = intracerebral hemorrhage* | |
